# Evaluation of HDPE and LDPE degradation by fungus, implemented by statistical optimization

**DOI:** 10.1038/srep39515

**Published:** 2017-01-04

**Authors:** Nupur Ojha, Neha Pradhan, Surjit Singh, Anil Barla, Anamika Shrivastava, Pradip Khatua, Vivek Rai, Sutapa Bose

**Affiliations:** 1 Earth and Environmental Science Research Laboratory, Department of Earth Sciences, Indian Institute of Science Education and Research Kolkata, Mohanpur 741246, Nadia, West Bengal, India; 2 Department of Physical Sciences, Indian Institute of Science Education and Research Kolkata, Mohanpur 741246, Nadia, West Bengal, India; 3 Institute of Life Sciences, Nalco Square, Bhubaneshwar, Odisha, India

## Abstract

Plastic in any form is a nuisance to the well-being of the environment. The ‘pestilence’ caused by it is mainly due to its non-degradable nature. With the industrial boom and the population explosion, the usage of plastic products has increased. A steady increase has been observed in the use of plastic products, and this has accelerated the pollution. Several attempts have been made to curb the problem at large by resorting to both chemical and biological methods. Chemical methods have only resulted in furthering the pollution by releasing toxic gases into the atmosphere; whereas; biological methods have been found to be eco-friendly however they are not cost effective. This paves the way for the current study where fungal isolates have been used to degrade polyethylene sheets (HDPE, LDPE). Two potential fungal strains, namely, *Penicillium oxalicum* NS4 (KU559906) and *Penicillium chrysogenum* NS10 (KU559907) had been isolated and identified to have plastic degrading abilities. Further, the growth medium for the strains was optimized with the help of RSM. The plastic sheets were subjected to treatment with microbial culture for 90 days. The extent of degradation was analyzed by, FE-SEM, AFM and FTIR. Morphological changes in the plastic sheet were determined.

Trillions of plastic bags are consumed each year^1^,^2^ and the used packaging materials are either dumped in landfills or degraded by using light, which is known as photo-degradation, by applying heat energy called as thermal degradation or by using microorganisms or biological additives, known as biodegradation^3^. A hefty figure of 140 million tonnes of synthetic polymers is produced on a global basis and their efficacy is increasing by the day^4^. Entire ecosystems are being destroyed as plastics in different forms are piled high in landfills and their reluctance in not being degraded by the extrinsic factors of nature, tags them as detestable. Although biodegradable plastic materials are on the rise, however, their usage has not been made popular and accessible to a large percentage of the society^5^.

High-density polyethylene and low density polyethylene are the long chain polymers of ethylene, which has been widely used in packaging industry due to its effectiveness and versatile nature such as light weight, inexpensive, durable, energy efficient and can be easily processed^6^,^7^,^8^,^9^.

The disposal of these used plastic materials by using chemical and physical methods are very expensive and produces persistent organic pollutants (POP’s) known as furans and dioxins, which are reported to be toxic irritant products, resulting into infertility of soil, preventing degradation of the other normal substances, depletion of the underground water source and have proved to be dangerous to animal, human and eco-system^10^. In addition, plastics are also reported to be biodegraded partially with the help of anaerobic process in composts and soil which produce carbon dioxide, water and methane^11^. However; the breakdown of large polymers to carbon dioxide (mineralization) requires several different organisms, with one breaking down the polymer into its constituent monomers, another one being able to use the monomers and excreting simpler waste compounds as by-products and a final one being able to use the excreted wastes^12^.

Thus, rapid biodegradation is the only eco-friendly process which can solve the problem faced by the plastic waste management efforts. In this process of biodegradation, plastic reacts with oxygen from the air and then the microorganisms, facilitate this degradation process by secreting polyethylene degrading enzymes to oxidize or break down the products for its energy into smaller byproducts such as carbon dioxide and water^13^. Although several microorganisms have been reported to degrade polyethylene, however the high molecular weight along with the three-dimensional structure and hydrophobic nature makes it averse towards degradation^14^,^15^. The hydrophobicity of polyethylene prevents the formation of bio-film by the microorganism which in return prevents the adhesion and colonization on the polyethylene.

A recent research article has suggested that the presence of groups such as, esterases, lipases and cutinases are usually responsible for degrading different forms of plastic^16^. A novel protein was also identified as ISF6_4381 secreted by the novel strain *Ideonella sakaiensis* 201-F6 was found to be responsible for the degradation of PET (Polyethylene terephthalate)^17^. Microorganisms such as bacteria for example *Brevibacillus borstelensis*^18^*, Rhodococcus rubber*^19^,^20^, as well as fungi (heterotrophic microorganisms) such as *Penicillium simplicissimum* YK^21^,^22^, *Fusarium solani*^23^ are reported to be used in degradation of both natural and synthetic polyethylene as its potential carbon substrate^24^,^25^,^26^. Efforts should be concentrated on developing eco-friendly methods of degrading synthetic plastics by utilizing the potential of microorganisms in degrading the various forms of plastics^27^,^28^,^29^.

The present study concentrates on isolating the potential fungal isolates responsible for the degradation of plastic from a soil sample collected from plastic dumping ground. The study also focuses on the part where response surface methodology has been used to optimize the growth media in order to increase the mycelium weight which would result into aiding the degradation of the HDPE and LDPE sheets. Several analytical methods have been used in a view to determine the extent of degradation of the plastic sheets (HDPE, LDPE).

## Materials and Methods

### Collection and processing of HDPE and LDPE sheets

HDPE and LDPE sheets were collected from department of Biological Sciences and Earth Science laboratories situated at IISER Kolkata, Mohanpur Campus, West Bengal. The sheets (test samples) were cut into small strips of 6 * 6 cm and transferred to a fresh solution containing 70 ml Tween 80, 10 ml bleach, and 983 ml distilled water and stirring for 30 to 60 minutes and sterilized as followed by El-Shafei *et al*.^27^ with a few modifications.

### Isolation and enrichment of fungal isolates

The soil sample was collected from a plastic dumping ground near IISER Kolkata, Mohanpur campus. The particular area has been chosen since it has been used to dump plastic for a very long time, increasing the probability of finding organisms that can degrade plastics. Isolation of the fungal colonies was done by using serial dilution method and spread plate technique on potato dextrose agar. Plates were incubated for 3–4 days at room temperature (28 °C) in the BOD incubator. The distinct fungal colonies were selected and subcultured for further characterization.

### Primary screening of potential fungal isolates

Primary screening of potential fungal isolates in both static and shaking condition was carried out in Czapek-Dox broth, at pH 7.03 ± 0.2, which was autoclaved and supplemented by sterilized HDPE, LDPE, a mixture of HDPE and LDPE sheets and by sucrose (30 gram/liter) which served as the control. Pure fungal cultures were isolated and inoculated separately and were incubated for 30 days, at room temperature for a static condition, and on the rotary shaker at 120 rpm for shaking condition. For negative controls, the following setups were used:

- Fungal inoculums + media (without carbon source).
- Media (without sucrose) + plastic (HDPE/LDPE).

Similarly, for positive controls, the following setups were used:

- Media (with sucrose) + inoculums + plastic (HDPE/LDPE).
- Media (with sucrose) + inoculums.

### Determination of the dry mycelium weight measurements

Fungal mycelium was filtered after 30 days of incubation and was vacuum dried for 24 hours. Mycelium weight for each flask was weighed by digital weighing balance and the fungal isolates with the highest mycelial weight were identified.

### Morphological Characterization and Phylogenetic analysis of the potential fungal isolates

The extraction of the DNA from the colonies and the PCR-DGGE analysis and the sequencing has been described in details in the supplementary section 1^29^. The sequence being novel was submitted to GenBank. For phylogenetic analysis, the fungal isolates’ ITS gene sequences from this work and other sequences retrieved from the database were aligned using the software CLUSTAL W 1.8^30^. The phylogenetical analysis was made using the neighbor-joining method. The analysis was performed with the software Bio NJ. The output trees were prepared using the software Tree view 1^31^,^32^.

### Optimization of Media with RSM

The growth media, Czapekdox broth, was optimized with the help of Response Surface Methodology. The compositions as well as the growth conditions such as the temperature and the pH were optimized and then the results were analyzed using the software, Design Expert version 7. The quadratic model was used to analyze the data. This particular model was best suited for the data since it allows the consideration of a number of constants in their model. The optimized media composition has been tabulated in supplementary table number 4.

### Mycodegradation analysis of HDPE and LDPE sheets

The potential pure fungal isolates were inoculated into each of the five sets of autoclaved flasks (500 ml), containing 100 ml of Czapekdox broth supplemented with sterilized HDPE, LDPE, and sucrose. The five inoculated sets were incubated at room temperature 28 °C in a static condition for a duration of 0, 15, 30, 45, 60 and 90 days, respectively.

### Determination of weight loss of the mycodegraded high and low density polyethylene by the potential fungal isolates

The dry weights of recovered HDPE and LDPE from the degradation media were taken in an interval of 30 days (i.e. day 0, day 30, day 60 and day 90) for accounting the rate of biodegradation. The HDPE and LDPE sheets were recovered by following the steps as discussed by Gilian *et al*.^18^, with some modifications. The weight difference between initial and final weight indicates the extent of polythene utilization by the fungi. Percentage weight loss was determined using the formula given in equation 1:


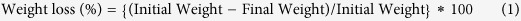


### Determination of total dry mycelium weight

After the incubation period of 0, 15, 30, 45, 60 and 90 days, each of the potential fungal mycelium was filtered separately by using vacuum filtration for individual flasks and were vacuum dried for a duration of 24 to 48 hours. Dried mycelial weight of individual fungal isolates (flasks for HDPE, LDPE, and Sucrose) was weighed by using digital weighing balance and the percentage of the fungal dry mycelium weights were calculated for each isolate with respect to their incubation periods.

### Characterization of the degraded HDPE and LDPE sheets

#### Fourier transform infrared spectroscopy (FTIR) analysis

The changes in the polymer bonds due to biodegradation were determined using FTIR spectrophotometer (Perkin Elmer, Spectrum RX1) and the Hydraulic Pellet Press (KP 799). The HDPE and LDPE sheets of different incubation periods (0, 15, 30, 45, 60 and 90 days) which were exposed to the test strains of fungi were analyzed. The sheets were cut into very small pieces and pellets were prepared with the help of KBr. The pellets were scanned in the region of 500–4000 cm^−1^ at a resolution of 1 cm^−1^ with the help of a single beam in the interferogram mode. Carbonyl index (CI) was used to measure the degree of biodegradation as its value depends on the degraded carbonyl bond. CI is obtained by the formula given in equation 2:


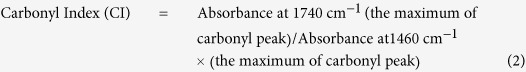


### Atomic force microscopy (AFM) analysis

The AFM was carried out by the model Agilent 6000 ILM AFM. The degraded LDPE and HDPE samples were prepared for Atomic force microscopy (AFM) along with their respective control films. All images were obtained with a scan speed of 1.0 Hz and a resolution of 512×512 pixels. The sample sheets were obtained from the broth where they were incubated for 90 days in presence of the two fungal isolates. The sample plastic sheets were washed with 2% sodium dodecyl sulfate to remove any mycobial debris from the broth. The plastic film was thereafter vacuum-dried for overnight and analyzed by AFM^33^.

### Scanning electron microscopy (FE-SEM) analysis

The changes in surface morphology of the LDPE and HDPE films, before and after biotic exposure were investigated using Field Emission Scanning Electron Microscope (Zeiss, Supra TM 55VP). The degraded LDPE and HDPE film samples were prepared for scanning electron microscopy (SEM) along with a control LDPE and HDPE film. The plastic strips were taken out after 0, 15, 30, 45, 60 and 90 days of incubation and were fixed with 2.5% gluteraldehyde in 0.05 M cacodylate buffer (1 h 30 min at 4 °C). The dehydrated samples were sputter-coated with a gold layer (Edwards S150B). The sputtering was achieved after passing the pure and dry argon gas in the coating chamber, under vacuum. The plate voltage was 2000 V and the current passed was 15 mA. A thickness of 2 nm of gold was achieved during a sputtering time of 10 s. The sample was then examined under the scanning electron microscope.

### Statistical Analysis

All the experiments were carried out in triplicates (n = 3) and the results are presented in mean value with standard deviation (Mean ± SD). The experiments were followed by completely randomized design (CRD) with three replicates for each treatment. All statistical analysis was performed with Graph Pad Prism software (version 5.03).

## Results

### Isolated fungal cultures from the dumping plastic site

A total of 10 fungal strains were isolated from the soil sample collected from the plastic dumping site. A total number of fungal colonies found on dilutions 10^−1^, 10^−2^, 10^−3^, 10^−4^, 10^−5^, 10^−6^, 10^−7^ and 10^−8^ on the potato dextrose agar plates are given in supplementary Table 1. The 10 isolated fungal strains were then labeled as NS1 through NS10. Supplementary Table 1, documents the total colony forming unit per gram of the isolated fungal cultures in each dilutions and the morphological characters of the 10 pure the fungal isolates is represented in supplementary Table 2.

### Primary screening of HDPE and LDPE degrading fungal isolates on solid medium

#### Determination of dry mycelium weight of the fungal isolates

**Figure 1:**
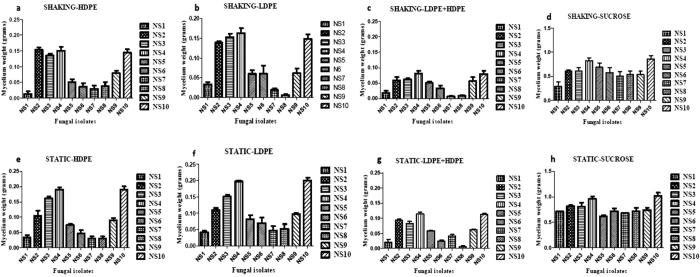
Dry mycelium weight determination post primary screening performed in shaking condition. Dry mycelium weight of the fungal isolates with the substrate HDPE sheets as a carbon source shown in (**a**), with LDPE sheets as a carbon source shown in (**b**), with HDPE and LDPE sheets as a carbon source shown in (**c**) and with the substrate sucrose as positive control shown in (**d**). Dry mycelium weight determination post primary screening performed in static condition. Dry mycelium weight of the fungal isolates with the substrate HDPE sheets as a carbon source shown in (**e**), with LDPE sheets as a carbon source shown in (**f**), with HDPE and LDPE sheets as a carbon source shown in (**g**) and with the substrate sucrose as positive control shown in (**h**).

Growth was observed in the flasks inoculated with fungal isolates named as NS4 and NS10 compared to the other cultures after 30 days of incubation in both the shaking and static conditions. The incubated flasks were filtered separately by using vacuum filter and the HDPE and LDPE degrading fungal isolates were screened by taking the dry mycelium and plastics’ weight. The comparative study was done in the following sequence, HDPE, LDPE, HDPE + LDPE and Sucrose, both in the static and shaking condition. It was observed that there was an improved growth in the static conditions for both the isolates. Hence, the rest of the experiment was carried out in the static condition itself. The results are represented in Fig. 1. A significance analysis was performed on the observed values also with the help of two-way ANOVA and it was found that in case of the two strains, the row factor, column factor, as well as the interaction, was found to be significant as the p value was <0.0001. As negative controls, the following setups were used:


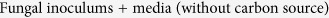


Post 30 days of incubation, a dry mycelium weight of 0.001 g was observed in the case of the isolates which possibly might have been due to the presence of other micronutrients in the medium. However, no major mycelium growth was seen in all the cases. This shows that the isolates are unable to grow in the absence of a carbon source.


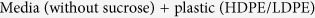


No reduction in the weight of the plastic was observed post 30 days of incubation. However, in the presence of the fungal strains, mycelium growth was observed which indicates that in the presence of the carbon source in the form of plastic, the fungal strains have been able to grow.

Similarly, as positive controls, the following setups were used:


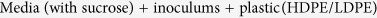


No reduction was observed post 30 days of incubation as the fungal isolates have been utilizing sucrose as the source of carbon as it is easier to break it down rather than plastic.


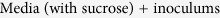


Maximum dry weight was observed among the isolates post 30 days of incubation.

### Phenotypic and molecular characterization of potential fungal isolates

**Figure 2:**
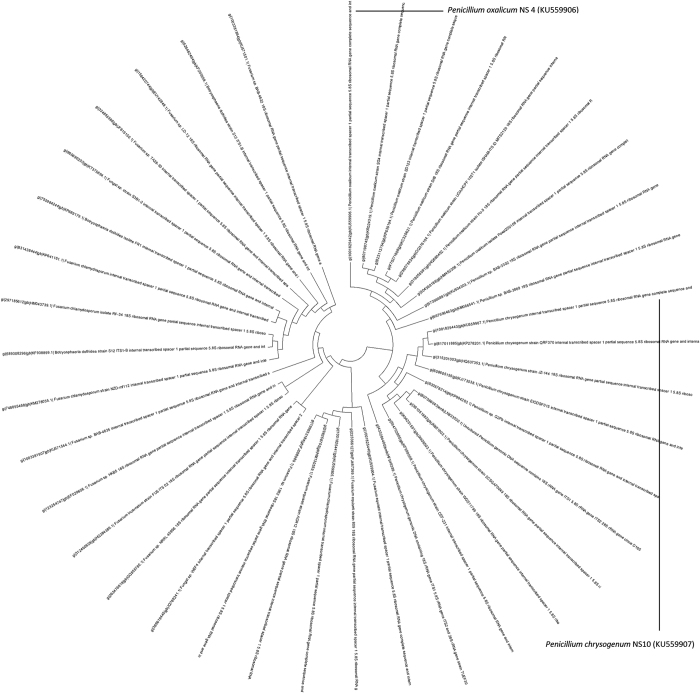
Representation of the phylogenetic tree with the novel strains. (**a**) *Penicillium chrysogenum* NS10 (KU55907) and (**b**) *Penicillium oxalicum* NS4 (KU55906).

The two potential fungal isolates were analyzed by ITS gene sequencing where DGGE was the preferred fingerprinting method. The BLAST search of the ITS sequence of the strain NS4 and NS10 showed the highest similarity with *Penicillium* and the phylogenetic tree was constructed using Tree View software. Based on the molecular taxonomy and phylogeny, the strains were designated as *Penicillium oxalicum* NS4 and *Penicillium chrysogenum* NS10, respectively. The nucleotide sequence data obtained during the study was submitted to NCBI under the accession numbers KU559906 for NS4 and KU559907 for NS10, respectively. The phylogenetic tree has been represented in Fig. 2 and the position of the two isolates has been shown, respectively. Supplementary Figure 4 accounts for the dry mycelium weight of NS4 and NS10 as they were subjected to growth conditions as the other isolates. Supplementary Figure 5 represents the microscopic image of NS4 and NS10 as stained by lactophenol cotton blue at 40X.

### Determination of weight loss of the HDPE and LDPE by the potential fungal isolates

**Figure 3:**
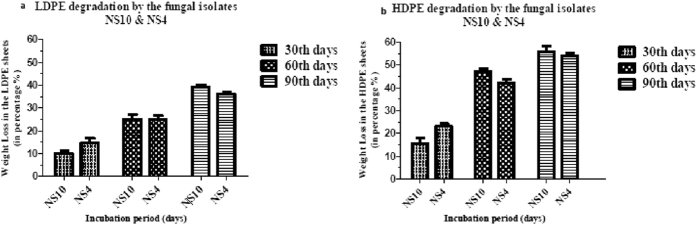
Measurement of the weight loss (in %) of the degraded (**A**) LDPE and (**B**) HDPE sheets by the potential isolates, namely, *Penicillium oxalicum* NS4 and *Penicillium chrysogenum* NS10 post 30, 60 and 90 days of incubation.

To quantify the HDPE and LDPE degradation efficiency of the two potential fungal isolates, the weight loss of the polyethylene films were measured after 90 days of incubation. The weight of HDPE sheets was reduced from 0.11244 to 0.09498, 0.07956, and 0.0598 and from 0.11233 to 0.08931, 0.07429, and 0.05897 after 30, 60 and 90 days of incubation with the fungal isolate NS10 and NS4, respectively. Similarly, the weight of LDPE sheets was reduced from 0.16443 to 0.13821, 0.12131, and 0.0954 and from 0.1649 to 0.13954, 0.12013, and 0.0989 after 30, 60 and 90 days of incubation with the fungal isolates NS10 and NS4, respectively. The control film showed no weight loss. Therefore, this result indicates that the weight loss of HDPE and LDPE during the incubation with the respective two isolates was due to the utilization of the polyethylene as the sole carbon source. The percentage of the total weight loss of the HDPE and LDPE sheets after 30, 60 and 90 days of incubation were found to be 17.06%, 48.00%, 58.598% and 19.32%, 33.33%, 34.35% by the fungal isolate NS10, respectively. Whereas, the total percentage of weight loss was found to be 24.18%, 43.73%, 55.34% in HDPE and 16.72%, 26.70%, 36.60% in LDPE sheets after 30, 60 and 90 days of incubation, by the fungal isolate NS4. The results indicate that both the isolates are capable of degrading HDPE quite efficiently which is more complicated than LDPE, which indicates that both the fungal isolates posses the ability to degrade plastic as shown in Fig. 3. ANOVA was performed on the data and it was found out that in the case of the two strains, the row factor, column factor, as well as the interaction, was found to be significant as the p value was <0.0001.

### Secondary Screening

#### RSM analysis

Response surface methodology is a collective unit of statistical analysis to optimize a system^34^. The system is theoretically designed and the results are optimized based on the experimental model suggested by the software. The Design Expert software has been considered to optimize the growth conditions of the two fungal isolates. Post analyses of the acquired data, two equations were derived based on the quadratic model used for optimization. The use of RSM results from the need of obtaining the optimal parameters supporting the growth of the fungal cultures and interprets interaction of the growth parameters with the variables. The equations are given below:

The quadratic model gave the following equation for the isolate NS4:

R1 = +0.90-0.045* A-0.025* B + 0.081* C + 5.712E-003* D + 0.043* E-0.035* F-0.076* G-0.071* H-0.10* A * B-0.14* A * C-0.11* A * D-0.026* A * E + 8.416E-003 * A * F + 0.027* A * G-0.027* A * H-0.039* B * C + 0.050* B * D-0.036* B * E-0.058* B * F-0.034* B * G + 4.713E-003* B * H + 0.026* C * D + 0.017* C * E + 0.14* C * F-0.054* C * G + 0.050* C * H-0.034* D * E-0.088* D * F-0.087* D * G + 0.061* D * H + 0.064* E * F-0.054* E * G + 0.036* E * H-5.307E-003* F * G-0.095* F * H + 0.068* G * H + 0.026* A^2^-0.23* B^2^-0.18* C^2^-0.047* D^2^ + 0.14* E^2^ + 0.060* F^2^-0.56* G^2^ + 0.23 * H^2^ (equation 2).

The quadratic model gave the following equation for the isolate NS10:

R1 = −38.46-3.39* A + 3.81* B + 3.60* C + 2.95* D + 1.52* E + 3.76* F + 5.13* G + 5.70* H-3.80* A * B-5.09* A * C-3.07* A * D-1.99* A * E-1.23* A * F-4.23* A * G-4.83* A * H + 4.23* B * C + 3.02* B * D-2.68* B * E + 7.50* B * F + 6.87* B * G + 6.29* B * H + 5.86* C * D-0.79* C * E + 3.40* C * F + 2.50* C * G + 7.67* C * H-2.38* D * E + 4.28* D * F + 3.74* D * G + 6.82* D * H-2.44* E * F-1.40* E * G-3.93* E * H + 3.96* F * G + 5.68* F * H + 7.78* G * H-2.12* A^2^+18.55* B^2^ + 1.17* C^2^+5.93* D^2^ + 5.61* E^2^ + 19.85* F^2^ + 8.26* G^2^-8.41* H^2^ (equation 3).

The F-test and ANOVA (Analysis of Variance) was carried out to determine the goodness of fit of the quadratic model. F-test measures the ratio of the variance between groups to the variance within groups and is represented by the F-value^34^. The models for both the NS4 and NS10 strains were observed to be significant because of the significance was seen in the F-value, adjusted R^2^ value, p-value and lack of fit. The model F-value of 3.69 in the case of NS4 and 3.63 in the case of NS10 was observed. There is only a 1.55% chance in case of NS4 and 1.65% chance in case of NS10, that a model F-value this large could occur due to noise. The Lack of Fit F-value of 0.35 for NS4 and 2.88 for NS10 implies that the Lack of Fit is not significant relative to the pure error. There is an 86.18% chance in case of NS4 and 13.52% chance in case of NS10, that a Lack of Fit value this large could occur due to noise. Non-significant Lack of Fit is good which only confirms the theory that the model is a good fit. Hegde *et al*.^34^ had made a similar observation in their work involving bacterial cellulose. In the case of the R^2^ value, the closer the value is to 1, the more significant it is. In the case of NS4, the adjusted R^2^ value is 0.9420, whereas in the case of NS10, the adjusted R^2^ value is 0.9411, which proves that a significant relationship exists between the independent variables and the responses obtained. A similar observation was noted by Rajasekhar *et al*.^35^. Normal plots represent how far the observed values have deviated from the predicted values obtained from the software-based analysis. Hence, it can be concluded that the statistical model indicated a 94.2% for NS4 and a 94.11% for NS10 of correlation between the predicted and the observed values from the normal plots.

**Figure 4:**
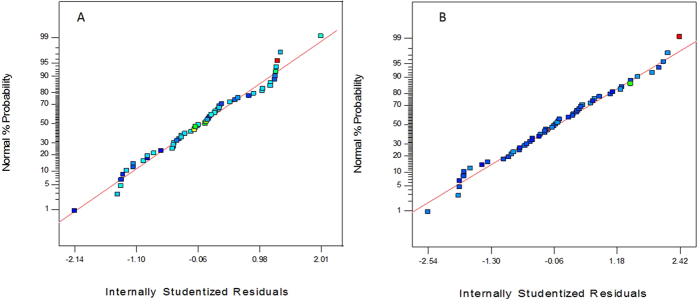
Normal Plots of Residuals for (**A**) *Penicillium oxalicum* NS4 and (**B**) *Penicillium chrysogenum* NS10.

The normal plots are represented in Fig. 4. The 3-D interactions between the different components of the media that were optimised to increase the growth of the strain *Penicillium oxalicum* NS4 and *Penicillium chrysogenum* NS10 has been shown in Supplementary Figures 1(A–D) and 2 (A–D). The optimized media details have been tabulated in supplementary Table 4.

### Determination of mycelium weight post optimization

The dry mycelium weight of the two potential fungal isolates, namely NS4 and NS10 were determined in the presence of the substrate LDPE, HDPE and Sucrose as their carbon and energy source, respectively after 90 days of degradation. The increase in the mycelium weight post optimization was significant. The observations have been tabulated in supplementary Table 5.

### Determination of the variation of the pH value of the medium

The variations in the pH value of the liquid medium containing LDPE, HDPE and sucrose (positive control) were determined by using pH meter and compared with its respective initial pH value after 90 days of incubation. Among the two substrates, the pH for the HDPE containing medium was seen to reduce gradually from 7.3 to 5 after 90 days of incubation, and it is represented in supplementary Figure 3. A significance analysis was performed on the observed values also with the help of two-way ANOVA, and it was seen that in case of the two strains, the row factor, column factor, as well as the interaction, was found to be significant as the p value was <0.0001.

### FTIR Analysis of the degraded HDPE and LDPE sheets

The sheet, which served the purpose of control, gave a peak at 3415.48 cm^−1^. A shift in the peak was observed both the times for the LDPE sheet treated with the microbial culture of *Penicillium chrysogenum* NS10 at 3436.85 cm^−1^ and 3430.45 cm^−1^ for the LDPE sheet treated with *Penicillium oxalicum* NS4. Sowmya *et al*.^36^ observed a similar shift in the peaks in her study involving degradation of polyethylene by the fungal consortium. In the case of the HDPE sheet, the control peak appeared at 3342 cm^−1^. A shift in the peak was observed both the times for the HDPE sheet, which was treated by the microbial culture *Penicillium chrysogenum* NS10 at 3423.33 cm^−1^ and 3370.50 cm^−1^ for the HDPE sheet treated with *Penicillium oxalicum* NS4. Bhatia *et al*.^37^ has carried out a similar work with a bacterial consortium, and a related shift in the peaks were observed. These peaks were observed due to the vibrations in the stretching of the O-H bond in alcohols and phenols. An absorbance range of 3500–3200 cm^−1^ corresponds to the presence of alcohols and phenols^37^.

The peak appeared at 2845 cm^−1^ for the LDPE control sheet and a shift in the peaks were observed in the LDPE sheet which was subjected to microbial treatment by *Penicillium chrysogenum* NS10 at 2898.66 cm^−1^ and at 2862.10 cm^−1^ for the sheet that was treated by *Penicillium oxalicum* NS4. Pramila and Ramesh^38^ carried out a similar study with *Acenitobacter baumannii* and its degradation effect on LDPE. In the case of the HDPE sheet, the peak appeared at 2821 cm^−1^ for the control sheet; however, a shift in the peak was observed at 2922.29 cm^−1^ when treated with NS10 microbial culture. A further shift in the peak was observed at 2877.05 cm^−1^ when the HDPE sheet was treated with NS4 fungal culture. Martinez-Romo *et al*.^39^ observed a similar shift in the peak on photodegradation of HDPE. The peaks were observed due to the vibrations in the stretching of the C = C bond seen in alkanes. Absorbance range of 3000–2800 cm^−1^ corresponds to C-H stretch and presence of alkanes^40^.

The peak appeared at 1445 cm^−1^ for the LDPE control sheet, a shift in the peak was observed at 1457.69 cm^−1^ in the sample treated by the microbial culture NS4. A further shift in the peak was observed at 1460.47 cm^−1^ on treatment with the microbial culture of NS10. Lal *et al*.^41^ had carried out a similar study where the LDPE sheet was subjected to photodegradation and a similar shift in the peak was observed. In case of the HDPE sheet, peak appeared at 1443 cm^−1^ for the control sheet and it had shifted to 1456.23 cm^−1^ when treated with NS4 and it was reduced to 1463.79 cm^−1^ when treated with NS10. Kowalczyk *et al*.^42^ carried out a similar study and a similar observation was made. Absorbance range of 1500–1400 cm^−1^ corresponds to −CH_2_ stretching and presence of aromatics^43^.

The peak appeared at 718 cm^−1^ for the HDPE control sheet, which had shifted to 719.30 cm^−1^ on treatment with the microbial culture NS4. On treatment with microbial culture NS10, a further shift in the peak appeared at 725.68 cm^−1^. In the case of LDPE, the peak appeared at 717 cm^−1^ for the control sheet and a shift in the peak appeared at 720.11 cm^−1^ for the LDPE sample treated with microbial culture NS4 and a further shift in the peak was observed at 729.73 cm^−1^ when treated with NS10. Ibiene *et al*.^44^ had carried out a similar study, on both HDPE and LDPE and similar shifts in the peaks were observed. The absorbance range of 700–900 cm^−1^ corresponds to −C = C− stretching and the presence of alkene group^45^.

**Figure 5:**
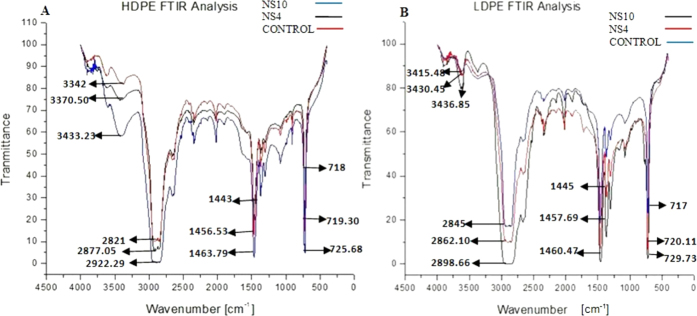
(**A**) Represents the peaks after the FTIR analysis of HDPE sheets post 90 days of incubation for NS4 and NS10 and 0^th^ day of incubation (control). (**B**) Represents the peaks after the FTIR analysis of LDPE sheets post 90 days of incubation for NS4 and NS10 and 0^th^ day of incubation (control).

The observed data has been represented in Fig. 5.

### Determination of the carbonyl index through FTIR analysis

**Figure 6:**
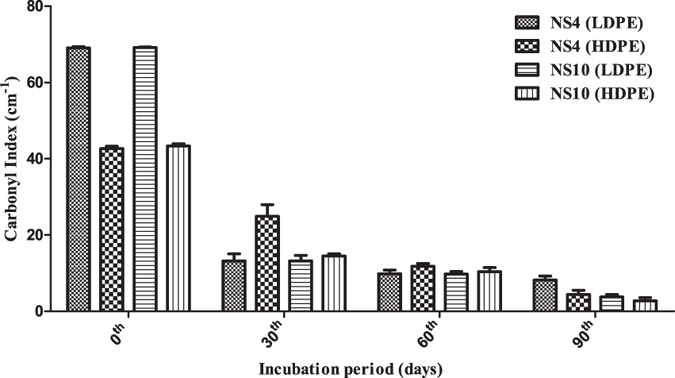
The plot represents the gradual decrease in the carbonyl indexes of the HDPE and LDPE sheets after 90 days of incubation with the microbial culture of *Penicillium oxalicum* NS4 and *Penicillium chrysogenum* NS10, respectively.

The carbonyl index of the low and high density polyethylene were calculated concerning their control and were found to be reducing gradually after 90 days of degradation. The decrease in the carbonyl index confirms the concept that oxidized polymers have been utilized by the microorganisms^46^. The carbonyl indexes as seen for the LDPE and HDPE sheets, when treated with microbial cultures of *Penicillium oxalicum* NS4 and *Penicillium chrysogenum* NS10, respectively has been tabulated in supplementary Table 3. The results have been represented in Fig. 6.

### Characterization of degraded HDPE & LDPE films by AFM analysis

**Figure 7:**
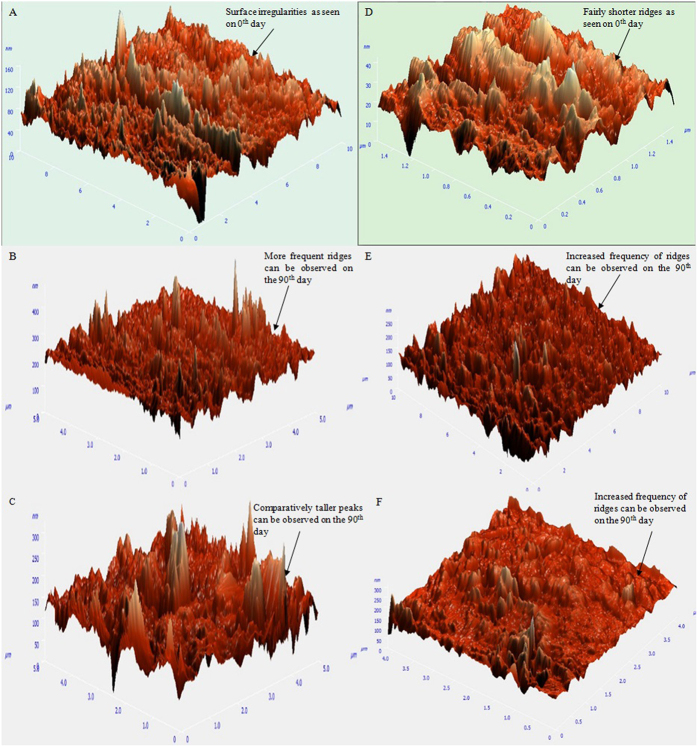
3D Atomic Force Microscopic view of the HDPE and LDPE degraded sheets. (**A**) HDPE sheet as seen on 0^th^ day (control). (**B**) HDPE sheet degraded by NS4 after 90 days of incubation. (**C**) HDPE sheet degraded by NS10 after 90 days of incubation. (**D**) LDPE sheet as seen on 0^th^ day (control). (**E**) LDPE sheet degraded by NS4 after 90 days of incubation. (**F**) LDPE sheet degraded by NS10 after 90 days of incubation.

AFM image analysis analyzed the surface morphology of the degraded HDPE and LDPE films, degraded by the *Penicillium oxalicum* NS4 and *Penicillium chrysogenum* NS10 strains. It was observed that the surface of the LDPE and HDPE film treated with the fungal isolates showed roughness, development of cracks and grooves. In contrast, the film that was not treated (control) with the isolates retained a smooth surface even after 90 days of incubation under the same condition. The morphological changes have been represented in Fig. 7 and the observed data has been tabulated in supplementary Table 6.

### Characterization of the degraded HDPE & LDPE films by FE-SEM analysis

**Figure 8:**
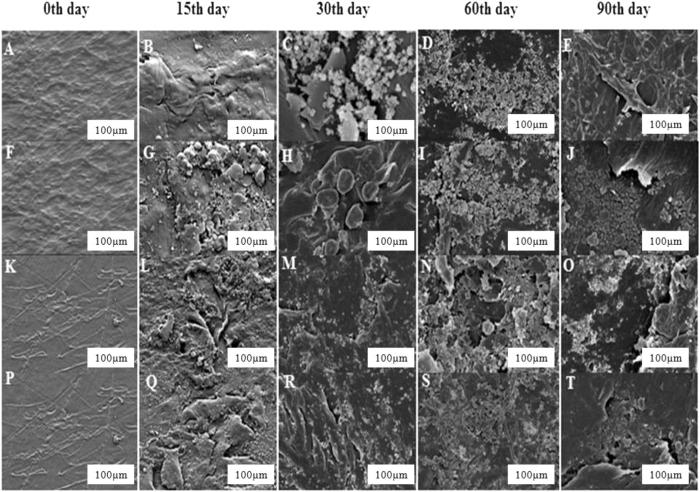
Field emission scanning electron microscopic view of the HDPE and LDPE degraded sheets. (**A**) HDPE sheet as seen on 0^th^ day (control). HDPE sheets as seen after (**B**) 15 days, (**C**) 30days, (**D**) 60 days and (**E**) 90 days of incubation with NS4. (**F**) HDPE sheet as seen on 0^th^ day (control). HDPE sheets as seen after (**G**) 15 days, (**H**) 30 days, (**I**) 60 days and (**J**) 90 days of incubation with NS10. (**K**) LDPE sheet as seen on 0^th^ day (control). LDPE sheets as seen after (**L**) 15 days, (**M**) 30 days, (**N**) 60 days and (**O**) 90 days of incubation with NS4. (**P**) LDPE sheet as seen on the 0^th^ day (control). LDPE sheets as seen after (**Q**) 15 days, (**R**) 30 days, (**S**) 60 days and (**T**) 90 days of incubation. 90 days of incubation with NS10.

To confirm the degradation of polyethylene by the fungal isolates, NS4 and NS10, the degraded sheets of HDPE and LDPE, were observed by scanning electron microscope. The surface morphology of the HDPE and LDPE films degraded by the *Penicillium oxalicum* NS4 and *Penicillium chrysogenum* NS10 strains were analyzed. In the case of NS4, attachment of the spores and mycelium network formation with the respective film were observed after 15 and 30 days of incubation. Few structural changes like grooves, cracks, damaged layer, fragileness, pits and roughening of the surface were observed after 45, 60 and 90 days of degradation. No apparent structural changes were found on the 0^th^ day or control films which were incubated under the same conditions. In the case of the films treated by the NS10, attachment of the spores was observed after 15 and 30 days of degradation. Bio-film formation and few structural changes like pits, grooves and cracks were observed on the degraded films after 45, 60 and 90 days of degradation. The results have been represented in Fig. 8.

## Discussion

Out of the ten fungal isolates, only NS4 and NS10 were found to show considerably higher growth rate as compared to the rest. NS4 and NS10 showed better growth in the static condition as compared to the shaking condition. The isolates were unable to grow in the absence of a carbon source and it confirmed the concept that only in the presence of the carbon source, which was plastic, the fungal strains have been able to grow. The phenotypic and molecular characterization determined the two strains to be as species of *Penicillium chrysogenum* and *Penicillium oxalicum.* ITS sequencing was carried out with the help of DGGE. The PCR amplified rDNA and the use of restriction endonucleases was applied to study the interspecies variation^47^,^48^. The secondary structures formed by the ITS is used for identification of cleavage sites and the binding sites for the RNA’s and nuclear proteins^48^. The variability of the 5.8 s rDNA region in fungi was studied with the help of ITS.

The growth conditions and media were subjected to optimization with the help of RSM to increase their vitality. The degradation rate of HDPE was determined as 55.598% by NS10 and 55.34% NS4 post 90 days of incubation. The degradation rate of LDPE was determined as 34.35% by NS10 and 36.60% NS4 post 90 days of incubation. The reduction in pH proves that the culture is still metabolically active and it is utilizing HDPE/LDPE for its growth concerning its positive control (media containing sucrose) which had been reduced to almost acidic pH. The reduction in pH not only confirms the usage of the polyethylene sheets as their source of carbon. However, it also paves the way for the idea regarding the production of several monomers which has possibly been produced post-degradation^48^.

The changes in bond scission, chemical transformation and formation and disappearance^49^ of new functional groups are the areas of interest that help us determine whether any changes in the chemical structure of the polyethylene has taken place or not^50^ was determined with the help of FTIR. As the rate of degradation increases with the passage of time, the peaks get broader as several monomeric and oxidative forms of the polyethylene gets produced^51^. The value of CI, which determines the extent of degradation, decreased with increase in incubation time and was observed to be the greatest for the two fungal isolates. The decrease in weight complemented the decrease in CI. Significance analysis such as two-way ANOVA was performed on the CI values and it was found to be significant since the p-value for the row value, column value, as well as the interaction, was found to be within limits (<0.0001).

Bonhomme *et al*.^51^ suggested in their study that AFM analysis of the plastic sheets can reveal pits or grooves, or morphological changes on the sheets caused due to the degradation by the fungal isolates. It is likely that these isolates produce unique enzymes which are capable of degrading polyethylene, and such enzymatic activities result in the formation of grooves. Since isolates can degrade LDPE/HDPE even without prior oxidation, it is possible that these species harbors enzyme(s) capable of oxidizing alkene bonds to carbonyls and carboxylic acids and thus eliminates the requirement of prior oxidation^52^. It is well known that the synthesis of bio-films by microorganisms favors their adhesion to the surface and help them to survive in a low nutrient environment and utilize solid substrates^53^. Bio-film reduces the hydrophobicity of the polymer and hence it improves the degradation rate^54^. Moreover, the hydrophobicity of the fungi would be facilitated in the absence of carbon source which in turn leads to increase in attachment properties of the fungi^55^. The bio-film formation presumably induced partial biodegradation. There were no structural changes observed on the 0^th^ day or control films which were incubated under the same conditions.

The FE-SEM images confirm that the two fungal strains have been able to break down the complex polymer of polyethylene of both HDPE and LDPE into its monomeric forms^56^. The grooves and cracks further confirm the fragility brought about to the plastic sheets on treating the sheets with fungal cultures. However, the comparison with the control sheet reinforces the degradation theory because they appear smooth on the 0^th^ day. The degradation is brought about by the formation of bio-film which has been suggested by Das and Kumar^57^. Only after the fungal isolates starts colonizing the plastic sheets by utilizing HDPE/LDPE as the sole source of carbon, the degradation commences.

There are several reports and findings available that suggest that the biodegradation of plastic or polyethylene takes place with the help of enzymes^55^. The reports encompass the prospect of both bacteria and fungi in this particular aspect. To discuss the scope of the same is similar in respect to this study. The existence of a similar kind of enzyme, as discussed by Koitabashi *et al*.^58^ is entirely possible for the *Penicillium sp.* Both Panagiotidou *et al*. and Sowmya *et al*. have studied about the enzymes responsible for degrading polyethylene. An example of enzymes as suggested by them includes the phylloplane fungal enzyme with respect to *Paraphoma* like phylloplane fungus^59^ and about the enzyme, PHB- depolymerase (3-Poly hydroxybutyrate) for *Penicillium pinophyllum*^59^. *Penicillium simplicissimum* is responsible for producing the polyethylene degrading enzymes, namely, laccase and manganese peroxidase^60^. PHB is extensively studied aliphatic polyester with an R configuration. Both bacteria and fungi have been observed to secrete PHB depolymerase which is responsible for hydrolyzing the ester bonds found in PHB and breaking it into oligomers and mono (3 hydroxybutyrate). With a molecular weight of 35 kDa, it binds to the surface of the polymer with the aid of the substrate binding domain. The products of the reaction are then further reduced to water and carbon dioxide. The fungi based PHB-decarboxylase has not been studied widely. Their study has been limited only to the two *Pencicillium* sp., namely, *Penicillium pinophilum* and *Penicillium*^59^. With a molecular weight of 35 kDa, it binds to the surface of the polymer with the aid of the substrate binding domain.

## Conclusion

The current study demonstrates the isolation, characterization and mycodegradation analysis of the degraded HDPE and LDPE films by the filamentous fast growing fungal strains that do not require prior oxidation or other chemical treatments. It indicates the presence of unique enzymatic activities in the two fungal strains. Apart from the enzymatic activities, the better degradation of LDPE and HDPE by the potential isolates can also be attributed to its ability to form a biofilm wherein the hydrophobicity of the cell surface may play a major role. Microbial colonization of synthetic plastic films is typically slow, which affects the total period of biodegradation. Gradual reduction, in the weight of the degraded HDPE and LDPE films concerning the time, signifies the utilization of these polymers as their source of nutrients as well as energy. There was an increase in the dry mycelium weight of all the four isolated fungal cultures when grown in the liquid medium containing HDPE and LDPE when optimized with the help of RSM.

Analysis through FE-SEM and AFM, of the degraded HDPE and LDPE films by the two strains showed morphological damages like pits, cracks, extensive roughening due to the formation of biofilm, grooves around the fungal cells, fragileness, fragmentations and erosion on the surface of the LDPE and HDPE sheets confirming that degradation had occurred by the action of the respective fungal isolates. FT-IR analysis of the degraded LDPE and HDPE films, showed the presence of proteinic materials, polysaccharides and metabolites produced by the fungal colonies which are major constituents of biofilms, alcohols, phenols, alkanes, amines and alkenes, being produced after 60 days, indicating that degradation was carried out successfully. Hence, the two potential fungal strains, namely *Penicillium oxalicum* NS4 and *Penicillium chrysogenum* NS10 can be said to be plastic degrading microorganisms.

The mechanism of degradation has been postulated to be under the presence of a series of enzymatic solubilization however the exact mechanism has not been fully understood. These findings support the previous work on biodegradation of LDPE and HDPE under natural environmental conditions although *in vitro* studies have not been thoroughly investigated. They also provide novel ways of implementing plant pathogen for degradation purpose over a long run. Thus the information procured acts as a shred of evidence for degradation capability of the isolated fungal organisms on LDPE and HDPE which can be further enhanced in an industrial scale for degrading various plastic materials.

## Additional Information

**How to cite this article**: Ojha, N. *et al*. Evaluation of HDPE and LDPE degradation by fungus, implemented by statistical optimization. *Sci. Rep.* **7**, 39515; doi: 10.1038/srep39515 (2017).

**Publisher's note:** Springer Nature remains neutral with regard to jurisdictional claims in published maps and institutional affiliations.

## Supplementary Material

Supplementary Dataset 1

## References

[b1] Caruso G. Plastic Degrading Microorganisms as a Tool for Bioremediation of Plastic Contamination in Aquatic Environments. J. Pollut. Eff. Cont. 3, 112 (2015).

[b2] Roy P. K. . Degradation of abiotically aged LDPE films containing pro-oxidant by bacterial consortium. Polym. Degrad. Stabil. 93(10), 1917–1922 (2008).

[b3] Indumathi A. & Gayathri T. Plastic Degrading ability of *Aspergillus oryzae* isolated from the garbage dumping sites of Thanjavur, India. Int. J. Curr. Microbiol. Appl. Sci. 8–13, 2319–7706 (2016).

[b4] Sekhar V. C. . Microbial degradation of high impact polystyrene (HIPS), an e-plastic with decabromodiphenyl oxide and antimony trioxide. J. Hazard. Mater. 318, 347–354 (2016). 27434738 10.1016/j.jhazmat.2016.07.008

[b5] Shimao M. Biodegradation of plastics. Curr. Opin. Biotechnol. 12, 242–247 (2001). 11404101 10.1016/s0958-1669(00)00206-8

[b6] Rivard C., Moens L., Roberts K., Brigham J. & Kelley S. Starch esters as biodegradable plastics: effect of ester group chain length and degree of substitution on anaerobic biodegradation. Enzyme Microb. Tech. 17, 848–852 (1995).

[b7] Begum M., Ariba B., Varalakshmi & Umamagheswari K.. Biodegradation of Polythene Bag using Bacteria Isolated from Soil. Int. J. Curr. Microbiol. App. Sci. 4(11), 674–680 (2015).

[b8] Witt U. . Biodegradation of aliphatic-aromatic copolyesters: evaluation of the final biodegradability and ecotoxicological impact of degradation intermediates. Chem. 44, 289–299 (2001). 10.1016/s0045-6535(00)00162-4 11444312

[b9] Muller R. J., Kleeberg I. & Deckwer W. D. Biodegradation of polyesters containing aromatic constituents. J. Biotechnol. 86, 87–95 (2001). 11245897 10.1016/s0168-1656(00)00407-7

[b10] Kyaw B. M., Champakalakshmi R., Sakharkar M. K., Lim C. S. & Sakharkar K. R. Biodegradation of low density polythene (LDPE) by Pseudomonas Species. Ind. J. Microbiol. 52(3), 411–419 (2012). 10.1007/s12088-012-0250-6 PMC3460136 23997333

[b11] Sumathi T., Vishwanath B., Laksmi A. S. & SaiGopal D. V. R. Production of Laccase by *Cochliobolus sp*. Isolated from Plastic Dumped Soils and Their Ability to Degrade Low Molecular Weight PVC. Biochem. Res. Int (2016). 10.1155/2016/9519527 PMC4880699 27293894

[b12] Sheik S., Chandrashekar K. R., Swaroop K. & Somashekarappa H. M. Biodegradation of gamma irradiated low density polyethylene and polypropylene by endophytic fungi. Int. Biodeter. Biodeg. 105, 21–29 (2015).

[b13] Albertsson A. C., Erlandsson B., Hakkareinen M. & Karlsson S. Molecular weight changes and polymeric matrix changes correlated with the formation of degradation products in biodegraded polyethylene. J. Env. Polym. Degr. 6, 187–195 (1998).

[b14] Ribitsch D. . Characterization of a new cutinase from *Thermobifida alba* for PET-surface hydrolysis. Biocatol. Biotransfor. 30, 2–9 (2012).

[b15] Yoshida S. . A bacterium that degrades and assimilates poly (ethylene terephthalate). Sci. 351(6278), 1196–1199 (2016). 10.1126/science.aad6359 26965627

[b16] Hadad D., Geresh S. & Sivan A. Biodegradation of polyethylene by the thermophilic bacterium *Brevibacillus borstelensis*. J. Appl. Microbiol. 98, 1093–1096 (2005). 15836478 10.1111/j.1365-2672.2005.02553.x

[b17] Sivan A., Szanto M. & Pavlov V. Biofilm development of the polyethylene degrading bacterium *Rhodococcus rubber*. Appl. Microbiol. Biotech. 72, 346–352 (2006). 10.1007/s00253-005-0259-4 16534612

[b18] Gilan I., Hadar Y. & Sivan A. Colonization, biofilm formation and biodegradation of polyethylene by a strain of *Rhodococcus rubber*. Appl. Microbiol. Biotechnol. 65, 97–104 (2004). 15221232 10.1007/s00253-004-1584-8

[b19] Yamada-Onodera K., Mukumoto Y., Katsuyaya Y., Saiganji A. & Tani Y. Degradation of polyethylene by a fungus *Penicillium simplicissimum* YK. Poly. Degrad Stabil. 72, 323–327 (2001).

[b20] Bharathidasan R. & Prince L. Biodegradation of low density polyethylene by bacteria from garbage soil in muthupet. Glob. J. Res. Anal. 5, 3 (2016).

[b21] Howard G. T. Biodegradation of polyurethane. Int. Biodeter. Biodegr. 49 (2002).

[b22] Glass J. E. & Swift G. Agricultural and synthetic polymers, biodegradation and utilization. ACS Symposium Series 433. American Chemical Society. Washington DC, 9–64 (1989).

[b23] Gu J. D., Ford T. E., Mitton D. B. & Mitchell R. Microbial corrosion of metals. 915–927 (New York, Wiley, 2001).

[b24] Devi R. S. . The Role of Microbes in Plastic Degradation. Environ.Waste Manage. 341 (2016).

[b25] Shah A. A., . Degradation of poly (ε-caprolactone) by a thermophilic bacterium *Ralstonia* sp. strain MRL-TL isolated from hot spring. Int. Biodeteri. Biodegr. 98, 35–42 (2015).

[b26] Dineshraj D. & Ganesh P.. Screening and Characterization of Isolated Fungi from Plastic Waste Dump Yard Sites. Int. J. Sci. Res. 5, 1 (2016).

[b27] El-Shafei H., El-Nasser N. H. A., Kansoh A. L. & Ali A. M. Biodegradation of disposable polyethylene by fungi *Streptomyces* species. Polym. Degrad. Stabil. 62, 361–365 (1998).

[b28] Anderson I. C., Campbell C. D. & Prosser J. I. Diversity of fungi in organic soils under a moorland- Scots pine (Pinus sylvestris L.) gradient. Environ. Microbiol. 5(11), 1121–1132 (2003). 14641592 10.1046/j.1462-2920.2003.00522.x

[b29] Deepika S. & Jaya M. R. Biodegradation of low density polyethylene by microorganisms from Garbage soil. J. Exp. Bio. Agri. Sci. 3(1), 15–21 (2015).

[b30] Gajendiran A., Krishnamoorthy S. & Abraham J. Microbial degradation of low-density polyethylene (LDPE) by *Aspergillus clavatus* strain JASK1.3 Biotech. 6(52) (2016). 10.1007/s13205-016-0394-x PMC4752946 28330123

[b31] Jeon H. J. & Kim M. N. Isolation of a thermophilic bacterium capable of low-molecular-weight polyethylene degradation. Biodegr. 24(1), 89–98 (2013). 10.1007/s10532-012-9560-y 22661062

[b32] Tribedi P. & Sil A. K. Low-density polyethylene degradation by *Pseudomonas* sp. AKS2 biofilm. Environ. Sci. Poll. Res. 20(6), 4146–4153 (2013). 10.1007/s11356-012-1378-y 23242625

[b33] Kumari R. . RSM Optimized media to increase the antibacterial activity of wild and mutated strain of *Nocardiopsis*VITSRTB. Res. J. Pharm. Tech. 7(2), 213–220 (2014).

[b34] Hegde S. . Statistical optimization of media components by Response Surface Methodology for enhanced production of bacterial cellulose by *Gluconacetobacter persimmonis*. J. Bioprocess. Biotech. 4(1) (2013).

[b35] Rajasekhar P., Ganesan G. & Senthilkumar C. Studies on tribiological behaviour of polyamide filled jute Fiber-Nano-ZnO hybrid composites. Procedia Eng. 97, 2099–2109 (2014).

[b36] Sowmya H. V., Ramalingappa B., Nayanashree G., Thippeswamy B. & Krishnappa M. Polyethylene degradation by fugal consortium. Int. J. Environ. Res. 9(3), 823–830 (2015).

[b37] Bhatia M., Girdhar A., Tiwari A. & Nayarisseri A. Implications of a novel Pseudomonas species on low density polyethylene biodegradation: an *in vitro* to in silico approach. Springer Plus. 3(497), (2014). 10.1186/2193-1801-3-497 PMC4409612 25932357

[b38] Pramila R. & Ramesh K. V. Potential biodegradation of low density polyethylene (LDPE) by Acenitobacter baumannii. African Journal of Bacteriology Research. 7(3), 24–28 (2015).

[b39] Martinez-Romo A., Mota R. G., Bernal J. J. S., Reyes C. F. & Candelas I. R. Effect of ultraviolet radiation in the photo-oxidation of high density polyethylene and biodegradable polyethylene films. J. Phy. Conference Series. 582 (2015).

[b40] Shah A. A., Hasan F., Hameed A. & Ahmed S. Isolation and characterization of poly (3-hydroxybutyrate-co-3-hydroxyvalerate) degrading bacteria and purification of PHBV depolymerase from newly isolated *Bacillus* sp. AF3. Int. Biodeter. Biodegr. 60, 109–115 (2007).

[b41] Lal V., Sikarwar A. & Paryani G. Photodegradation of LDPE films: Statistical analysis with Carbonyl Index. Asian J. Biochem. Pharm. Res. 4(4), 203–209 (2014).

[b42] Kowalczyk A., Chyc M., Ryszka P. & Latowski D. *Achromobacter xylosodixans* as a new microorganism strain colonizing high-density polyethylene as a key step to its biodegradation. Environ. Sci. Poll. Res. 23, 11349–11356 (2016). 10.1007/s11356-016-6563-y PMC4884572 27072033

[b43] Negi. H., Gupta S., Zaidi M. G. H. & Goel R. Studies on biodegradation of LDPE film in the presence of potential bacterial consortia enriched soil. Biologia. 57, 141–147 (2011).

[b44] Ibiene A. A., Stanley H. O. & Immanuel O. M. Biodegradation of polyethylene by *Bacillus sp.* indigenous to the Niger Delta mangrove swamp. Nigeria. J. Biotech. 26, 68–79 (2013).

[b45] Esmaeili A., Pourbabaee A. A., Alikhani H. A., Shabani F. & Kumar L. Colonization and Biodegradation of Photo-Oxidized Low-Density Polyethylene (LDPE) by New Strains of *Aspergillus* sp. and *Lysinibacillus* sp. Biorem. J. 18(3), 213–226 (2014).

[b46] Ratanakamnuan U. & Aht-ong D. Photobiodegradation of low-density polyethylene/banana starch films. J. Appl. Polym. Sci. 100, 2725–2736 (2006).

[b47] Meenakshi P. . Mechanical and microstructure studies on the modification of the CA film by blending with PS. Bull. Mater. Sci. 25(1), 25–29(2002).

[b48] Arutchelvi J. . Biodegrdation of polyethylene and polypropylene. Ind. J. Biotech. 7, 9–22 (2008).

[b49] Usha R., Sangeetha T. & Palaniswamy M. Screening of polyethylene degrading microorganisms from garbage soil. Libyan Agri. Res. Cen. J. Int. 2(4), 200–204 (2011).

[b50] Suresh B. . Influence of thermal oxidation on surface and thermo-mechanical properties of polyethylene. Journal of Polymer Research. 18(6), 2175–2184 (2011).

[b51] Bonhomme S. . Environmental biodegradation of polyethylene. Polym. Degrad. Stabil. 81, 441–452 (2003).

[b52] Yoon M. G., Jeon H. J. & Kim N. M. Biodegradation of Polyethylene by a soil bacterium and *Alk B* cloned recombinant cell. J. Bioremed. Biodegrad. 3(4), 1–8 (2012).

[b53] Maquelin K. . Microbiol. Methods. 51, 255–71 (2002). 10.1016/s0167-7012(02)00127-6 12223286

[b54] Kiatkamjornwong S., Thakeow P. & Sonsuk M. Chemical modification of cassava starch for degradable polyethylene sheets. Polym. Degrad. Stabil. 73(2), 363–375 (2001).

[b55] Raaman N., Rajitha N., Jayshree A. & Jegadeesh R. Biodegradation of plastic by Aspergillus sp. isolated from polythene polluted sites around Chennai. J. Acad. Indus. Res. 1(6), 313–316 (2012).

[b56] Sanin S. L., Sanin F. D. & Bryers J. D. Effect of starvation on the adhesive properties of xenobiotic degrading bacteria. ProcessBiochem. 38(6), 909–914 (2003).

[b57] Das M. P. & Kumar S. Microbial deterioration of Low Density Polyethylene by *Aspergillus* and *Fusarium sp.* Int. J. ChemTech Res. 6(1), 299–305 (2014).

[b58] Koitabashi M., Sameshima-Yamashita Y., Watanabe T., Shinozaki Y. & Kitamoto H. Phylloplane Fungal Enzyme Accelerate Decomposition of Biodegradable Plastic Film in Agricultural Settings. Japan Agri. Res. Quart. 50(3), 229–234 (2016).

[b59] Panagiotidou E. . Simple Route for Purifying Extracellular Poly (3-hydroxybutyrate)-depolymerase from *Penicillium pinophilum*. Enzyme Res. Article ID 159809, 6 pages (2014). 10.1155/2014/159809 PMC4190121 25328684

[b60] Santo M., Weitsman R. & Sivan A. The role of the copper-binding enzyme-laccase-in the biodegradation of polyethylene by the actinomycete *Rhodococcus ruber*. Int. Biodeter. Biodegr. 84, 204–210 (2013).

