# Supplementary material for: Evaluation of HDPE and LDPE degradation by fungus, implemented by statistical optimization
Source: Sci Rep. 2017 Jan 4;7:39515. doi: 10.1038/srep39515 (PMC5209683; doi:10.1038/srep39515)
Supplement: Supplementary Dataset 1 [file srep39515-s1.doc]

**SUPPLEMENTARY**

**Evaluation of HDPE and LDPE degradation by fungus, implemented by statistical optimization**

**Nupur Ojha^Ψ1^, Neha Pradhan^Ψ1^, Surjit Singh^1^, Anil Barla^1^, Anamika Shrivastava^1^, Pradip Khatua^2^, Vivek Rai^3*^, Sutapa Bose^1*^**

**¥** Authors have contributed equally to this work.

1. Earth and Environmental Science Research Laboratory, Department of Earth Sciences, Indian Institute of Science Education and Research Kolkata, West Bengal, India
2. Department of Physical Sciences, Indian Institute of Science Education and Research Kolkata, Mohanpur 741246, Nadia, West Bengal, India
3. Institute of Life Sciences, Nalco Square, Bhuvaneshwar, Odisha, india.

*Corresponding Authors (Tel.: + 91- 8145283082; E mail: [sutaparai@gmail.com](mailto:sutaparai@gmail.com), [sutapa.bose@iiserkol.ac.in](mailto:sutapa.bose@iiserkol.ac.in))

(Tel: +91-7894368888, Email: [vivekrai.a@gmail.com](mailto:vivekrai.a@gmail.com) and [vivek.rai@ils.res.in](mailto:vivek.rai@ils.res.in) )

Table of Contents

[Supplementary section 1. 2](#__RefHeading___Toc464419967)

[Isolation of DNA 3](#__RefHeading___Toc464419968)

[PCR-DGGE analysis 3](#__RefHeading___Toc464419970)

[Supplementary Figure 1A. 4](#__RefHeading___Toc464419971)

[Supplementary Figure 1B. 5](#__RefHeading___Toc464419972)

[Supplementary Figure 1C 6](#__RefHeading___Toc464419973)

[Supplementary Figure 1D 7](#__RefHeading___Toc464419974)

[Supplementary Figure 2A. 8](#__RefHeading___Toc464419975)

[Supplementary Figure 2B. 9](#__RefHeading___Toc464419976)

[Supplementary Figure 2C. 10](#__RefHeading___Toc464419977)

[Supplementary Figure 2D. 11](#__RefHeading___Toc464419978)

[Supplementary Figure 3 12](#__RefHeading___Toc464419979)

[Supplementary Figure 4 13](#__RefHeading___Toc464419980)

[Supplementary Figure 5 14](#__RefHeading___Toc464419981)

[Supplementary Table1. 15](#__RefHeading___Toc464419983)

[Supplementary Table 2. 16](#__RefHeading___Toc464419984)

[Supplementary Table3. 17](#__RefHeading___Toc464419985)

[Supplementary Table4. 18](#__RefHeading___Toc464419986)

[Supplementary Table5. 19](#__RefHeading___Toc464419987)

[Supplementary Table6. 20](#__RefHeading___Toc464419988)

# Supplementary section 1.

# Isolation of DNA

# Fungal cells were obtained from the colony which was washed using sterile water and later incubated in 100 µl of lyticase solution at 30 ºC for 1 hr. The suspension was further incubated at 55 ºC for 9 min after an addition 20 µl. PCR reaction mixtures of 10 µl was prepared post boiling the mixture for 8 min.

# PCR-DGGE analysis

The primers ITS1 (5´TCC GTA GGT GAA CCTTGC GG 3´) and ITS4 (5´TCC TCC GCT TAT TGA TAT GC 3´) were used (Anderson *et al*., 2003). PCR reaction mixture contained 0.5 µM of each primer, 10 µM of deoxynucleotides, 1.5 mM MgCl_2_ and 1x buffer (Sigma-Aldrich). A thermocycler was used to heat the reaction mixture at 95 ºC for 15 min. Taq polymerase was added to the reaction mixture and inserted in the PCR machine with the following setup:

98 ºC- 5 min, 98 ºC -30 sec, 55 ºC - 45 sec, 72 ºC – 2 min, 72 ºC 10 min for 35 cycles.

The products were further digested with restriction endonucleases, CfoI, HaeIII and Hinfl. The restricted fragments of the PCR products were separated on agarose gel.


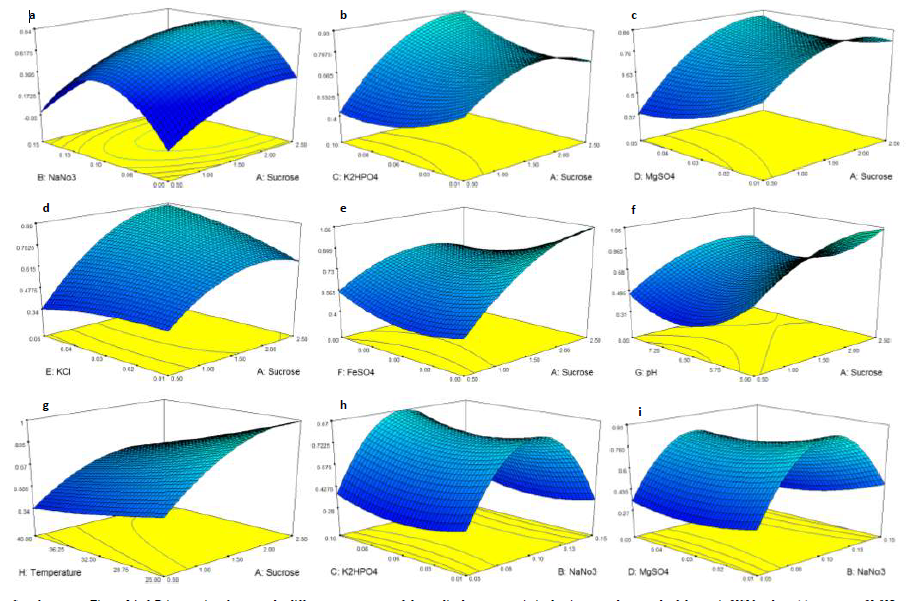


Supplementary Figure 1A. **3-D interactions between the different components of the media that were optimised to increase the growth of the strain NS10, where (a) represents NaNO_3_ vs Sucrose, (b) K_2_HPO_4_ vs Sucrose, (c) MgSO_4_ vs Sucrose, (d) KCl vs Sucrose, (e) FeSO_4_ vs Sucrose, (f) pH vs Sucrose, (g) Temperature vs Sucrose, (h) K_2_HPO_4_ vs NaNO_3_, (i) MgSO_4_ vs NaNO_3._**

**
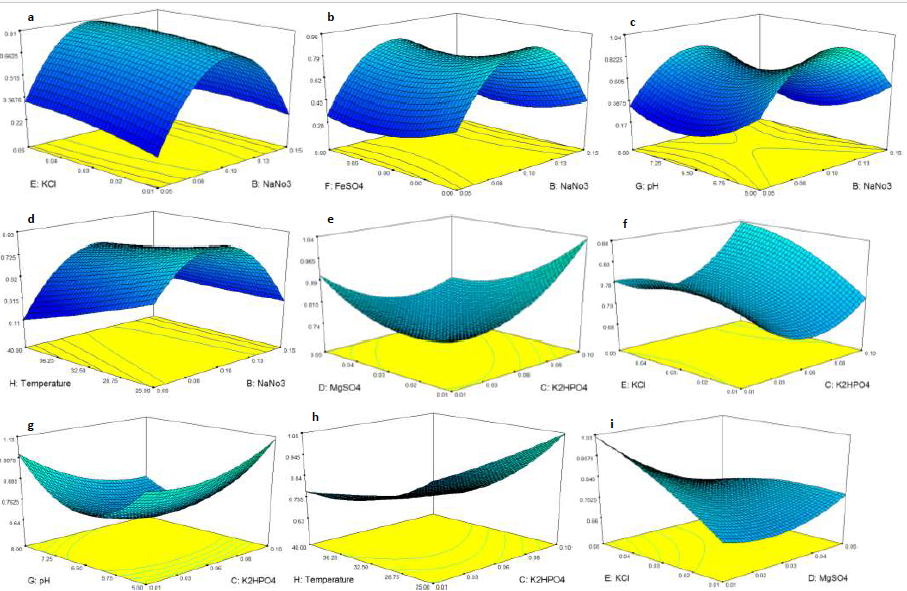
**

Supplementary Figure 1B. **3-D interactions between the different components of the media that were optimised to increase the growth of the strain NS10, where (a) KCl vs NaNO_3_, (b) FeSO_4_ vs NaNO3, (c) pH vs NaNO_3_, (d) Temperature vs NaNO_3_, (e), (o) MgSO_4_ vs K_2_HPO_4_, (f) KCl vs K_2_HPO_4_, (g) pH vs K_2_HPO_4_, (h) Temperature vs K_2_HPO_4_, (i) KCl vs MgSO_4_**

**
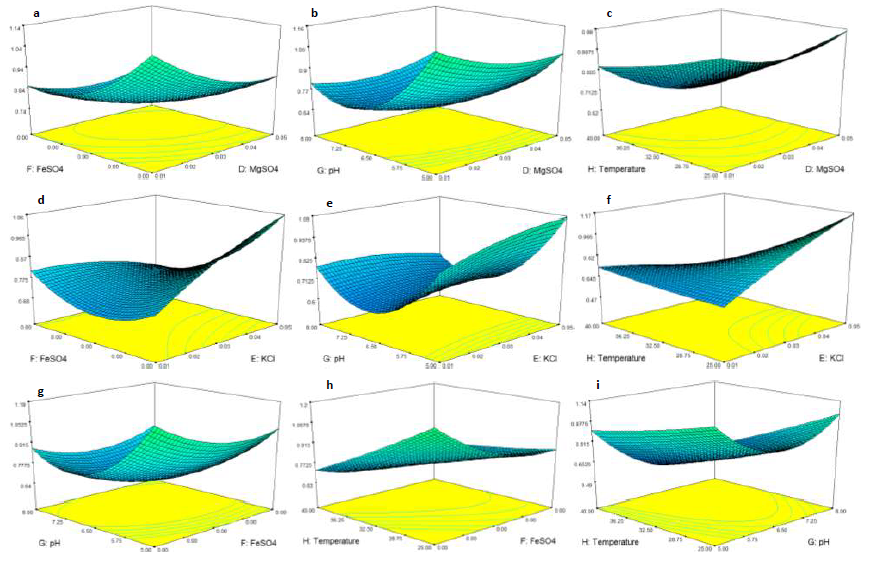
**

Supplementary Figure 1C**. 3-D interactions between the different components of the media that were optimised to increase the growth of the strain NS10, where (a) FeSO_4_ vs MgSO_4_, (b) pH vs MgSO_4_, (c) Temperature vs MgSO_4_, (d)FeSO_4_ vs KCl, (e) pH vs KCl, (f) Temperature vs KCl, (g) pH vs FeSO_4_, (h) Temperature vs FeSO_4_, (i) Temperature vs pH.**

**
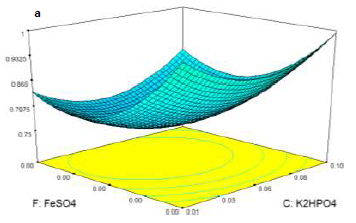
**

Supplementary Figure 1D**. 3-D interactions between the different components of the media that were optimised to increase the growth of the strain NS10, where (a) represents FeSO_4_ vs K_2_HPO_4._**


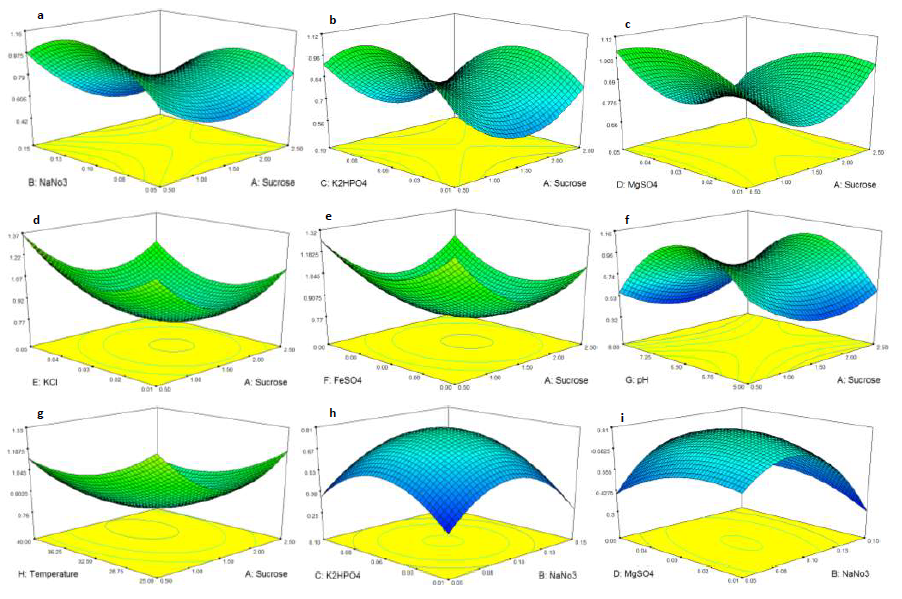


Supplementary Figure 2A. **3-D interactions between the different components of the media that were optimised to increase the growth of the strain NS4, where (a) represents NaNO_3_ vs Sucrose, (b) K_2_HPO_4_ vs Sucrose, (c) MgSO_4_ vs Sucrose, (d) KCl vs Sucrose, (e) FeSO_4_ vs Sucrose, (f) pH vs Sucrose, (g) Temperature vs Sucrose, (h) K_2_HPO_4_ vs NaNO_3_, (i) MgSO_4_ vs NaNO_3_**

**
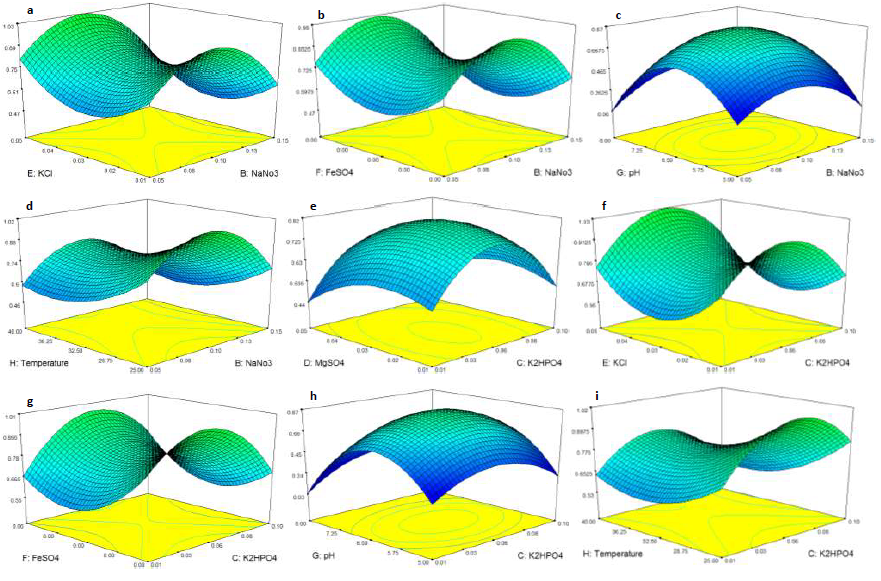
**

Supplementary Figure 2B. **3-D interactions between the different components of the media that were optimised to increase the growth of the strain NS4, where (a) represents KCl vs NaNO_3_, (b) FeSO_4_ vs NaNO_3_, (c) pH vs NaNO_3_, (d) Temperature vs NaNO_3_, (e) MgSO_4_ vs K_2_HPO_4_, (f) KCl vs K_2_HPO_4_, (g) FeSO_4_ vs K_2_HPO_4_, (h) pH vs K_2_HPO_4_, (i) Temperature vs K_2_HPO_4._**

**
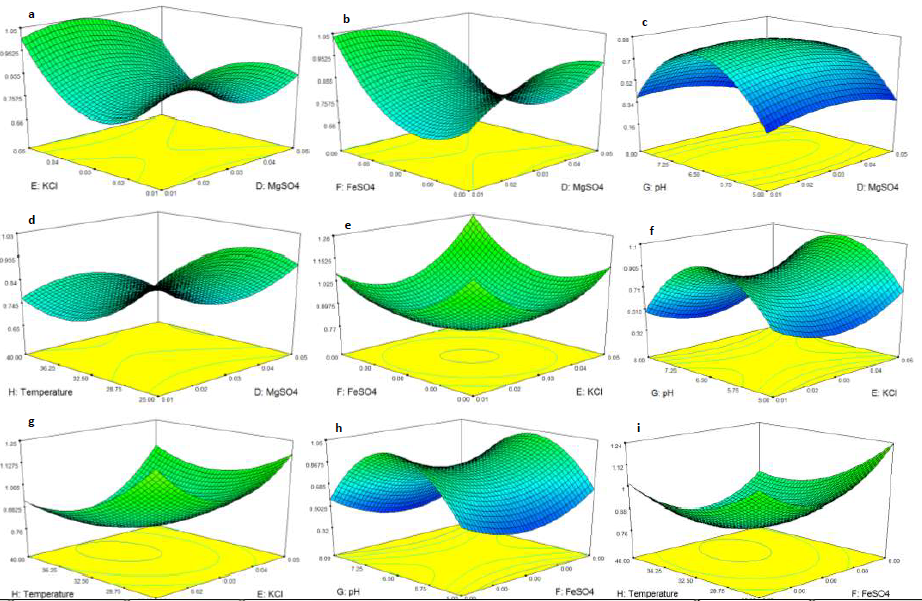
**

Supplementary Figure 2C. **3-D interactions between the different components of the media that were optimised to increase the growth of the strain NS4, where (a) represents KCl vs MgSO_4_, (b) FeSO_4_ vs MgSO_4_, (c) pH vs MgSO_4_, (d) Temperature vs MgSO_4_, (e) FeSO_4_ vs KCl, (f) pH vs KCl, (g) Temperature vs KCl, (h) pH vs FeSO_4_, (i) Temperature vs FeSO_4._**

**
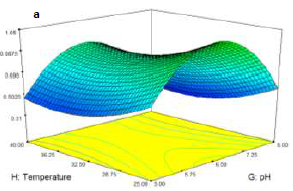
**

Supplementary Figure 2D. **3-D interactions between the different components of the media that were optimised to increase the growth of the strain NS4, where (a) represents Temperature vs pH.**


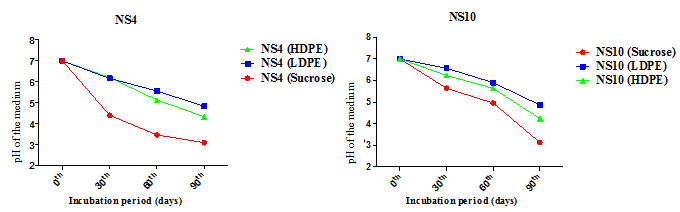


Supplementary Figure 3. **Variation in the pH value of the medium as seen after 90 days where the potential fungal isolates, *Penicilium oxalicum* NS4 (A) and *Penicillium chrysogenum* NS10 (B), were grown in the presence of HDPE, and LDPE as a substrate after 30 days of time interval with respect to their positive control, sucrose.**

**
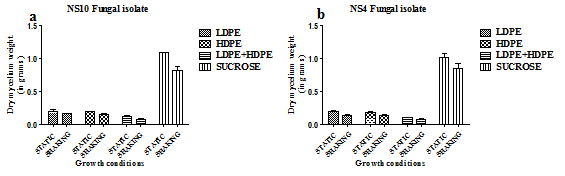
**

Supplementary Figure 4**. Dry mycelium weight determination for fungal isolates (a) NS10 and (b) NS4 for LDPE, HDPE, HDPE+LDPE and sucrose based growth conditions in both the static and the shaking, respectively.**

**
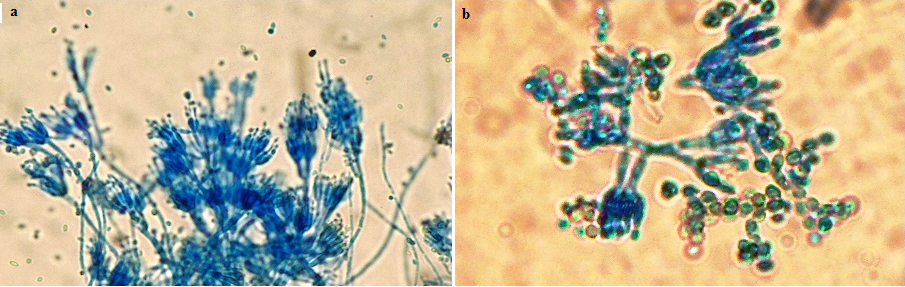
**

Supplementary Figure 5**. Microscopic representation of the fungal isolates (a) NS4 and (b) NS10 stained with the help of lactophenol cotton blue and observed under 40X.**

# **Supplementary Table1.** The total colony forming unit per gram of the isolated fungal cultures in each dilutions is represented

| **Dilution factor** | **Total number of colonies** | **Colony forming unit per gram by using the formula: total no. of colonies/dilution factor * volume** |
| --- | --- | --- |
| **10^-1^** | 32 | 32/(0.1*10^-1^) = 3.2*10^-3^ CFU/gram |
| **10^-2^** | 28 | 28/(0.1*10^-2^) = 28*10^-3^ CFU/gram |
| **10^-3^** | 18 | 18/(0.1*10^-3^) = 180*10^-3^CFU/gram |
| **10^-4^** | 14 | 14/(0.1*10^-4^) = 1,400*10^-3^CFU/gram |
| **10^-5^** | 10 | 10/(0.1*10^-5^) = 10,000*10^-3^CFU/gram |
| **10^-6^** | 8 | 8/(0.1*10^-6^) = 80,000*10^-3^CFU/gram |
| **10^-7^** | 5 | 5/(0.1*10^-7^) = 500,000*10^-3^CFU/gram |
| **10^-8^** | 3 | 3/(0.1*10^-8^) = 3,000,000*10^-3^ CFU/gram |
| **Average** |  | Total CFU/no. of dilution factors  =448,950.6*10^-3^CFU/gram |

Supplementary Table 2.**Morphological characterization of the pure fungal isolates NS1 through NS10.**

| **S.No** | **Fungal**  **Isolates** | **Form** | **Elevation** | **Margin** | **Color** | **Type** |
| --- | --- | --- | --- | --- | --- | --- |
| **1.** | NS1 | Filamentous | Umbonate | Erose | White | Cottony |
| **2.** | NS2 | Filamentous | Umbonate | Filamentous | Yellowish white | Bushy |
| **3.** | NS 3 | Filamentous | Umbonate | Filamentous | Reddish white | Cottony |
| **4.** | NS 4 | Irregular | Umbonate | Erose | Whitish with greenish edge | Whitish center with greenish edge |
| **5.** | NS 5 | Circular | Pulvinate | Entire | Green | Velvety |
| **6.** | NS 6 | Circular | Pulvinate | Entire | Dark green | Velvety |
| **7.** | NS 7 | Irregular | Umbonate | Undulate | Green | Velvety |
| **8.** | NS 8 | Irregular | Umbonate | Erose | Brownish green | Bushy greenish |
| **9.** | NS 9 | Irregular | Umbonate | Undulate | Dark green | Velvety |
| **10.** | NS 10 | Irregular | Pulvinate | Entire | Yellowish green | Velvety |

Supplementary Table 3. **The carbonyl indexes as seen for the LDPE and HDPE sheets when treated with microbial cultures of *Penicillium oxalicum* NS4 and *Penicillium chrysogenum* NS10 respectively.**

| **No. of Days** | **NS4 (LDPE)** | | | | **NS4 (HDPE)** | | | | **NS10 (LDPE)** | | | | **NS10 (HDPE)** | | | |
| --- | --- | --- | --- | --- | --- | --- | --- | --- | --- | --- | --- | --- | --- | --- | --- | --- |
| **0^th^** | 69.220 | 69.220 | 68.660 | | 42.987 | 42.987 | | 41.897 | 69.220 | | 69.220 | 68.898 | 43.987 | | 42.987 | 42.987 |
| **30^th^** | 11.622 | 15.224 | 12.781 | | 27.330 | 21.421 | | 25.879 | 14.701 | | 13.057 | 11.897 | 13.879 | | 14.624 | 15.027 |
| **60^th^** | 8.932 | 10.792 | 9.785 | | 10.877 | 12.366 | | 11.984 | 9.877 | | 10.356 | 8.987 | 10.786 | | 9.205 | 11.202 |
| **90^th^** | 9.205 | 8.282 | 7.086 | | 3.070 | 5.312 | | 4.678 | 4.282 | | 3.089 | 3.872 | 2.733 | | 3.546 | 1.911 |
|  | | | |  | | |  | | |  | |  | |  | | |
|  | | | |  | | |  | | |  | |  | |  | | |
|  | | | |  | | |  | | |  | |  | |  | | |
|  | | | |  | | |  | | |  | |  | |  | | |
|  | | | |  | | |  | | |  | |  | |  | | |
|  | | | |  | | |  | | |  | |  | |  | | |
|  | | | |  | | |  | | |  | |  | |  | | |
|  | | | |  | | |  | | |  | |  | |  | | |
|  | | | |  | | |  | | |  | |  | |  | | |
|  | | | |  | | |  | | |  | |  | |  | | |
|  | | | |  | | |  | | |  | |  | |  | | |
|  | | | |  | | |  | | |  | |  | |  | | |
|  | | | |  | | |  | | |  | |  | |  | | |
|  | | | |  | | |  | | |  | |  | |  | | |
|  | | | |  | | |  | | |  | |  | |  | | |
|  | | | |  | | |  | | |  | |  | |  | | |
|  | | | |  | | |  | | |  | |  | |  | | |
|  | | | |  | | |  | | |  | |  | |  | | |
|  | | | |  | | |  | | |  | |  | |  | | |
|  | | | |  | | |  | | |  | |  | |  | | |
|  | | | |  | | |  | | |  | |  | |  | | |

Supplementary Table 4. **The basic and optimized composition of the Czapekdox agar**

| **Components of the media** | **Basic (50ml)** | **Optimized (50 ml)** |
| --- | --- | --- |
| Sucrose | 1.5 | 2.5 |
| NaNO_3_ | 0.1 | 0.05 |
| K_2_HPO_4_ | 0.05 | 0.06 |
| MgSO_4_ | 0.025 | 0.05 |
| KCl | 0.025 | 0.01 |
| FeSO_4_ | 0.0005 | 0.0005 |

Supplementary Table 5. **Dry mycelium weight in gram of the potential isolates grown in the presence of HDPE, LDPE and Sucrose (+C) as a substrate is shown post optimization of the growth media.**

| No. of days | F4 | | | F10 | | |
| --- | --- | --- | --- | --- | --- | --- |
|  | **L** | **H** | **+ C** | **L** | **H** | **+ C** |
| 0^th^ | 0.0 | 0.0 | 0.0 | 0.0 | 0.0 | 0.0 |
| 30^th^ | 0.030 | 0.028 | 0.165 | 0.038 | 0.023 | 0.138 |
| 45^th^ | 0.043 | 0.042 | 0.252 | 0.037 | 0.036 | 0.213 |
| 60^th^ | 0.0473 | 0.051 | 0.276 | 0.0446 | 0.085 | 0.256 |
| 90^th^ | 0.034 | 0.045 | 0.270 | 0.0433 | 0.084 | 0.269 |

**Where, “L, H and +C” signifies for LDPE, HDPE and positive control respectively**.

Supplementary Table 6. **Increment in the roughness observed on the surface of the degraded HDPE and LDPE sheets after 90days.**

| **Fungal isolates** | HDPE | | LDPE | |
| --- | --- | --- | --- | --- |
|  | **0^th^ day** | **90^th^ day** | **0^th^ day** | **90^th^ day** |
| ***Penicillium oxalicum* NS4** | 4.3371 nm | 47.576 nm | 9.137 nm | 47.236 nm |
| ***Penicillium chrysogenum* NS10** | 4.3371 nm | 44.516 nm | 9.137 nm | 34.716 nm |
